# Plant-Based Vaccines: The Way Ahead?

**DOI:** 10.3390/v13010005

**Published:** 2020-12-22

**Authors:** Zacharie LeBlanc, Peter Waterhouse, Julia Bally

**Affiliations:** 1 Centre for Agriculture and the Bioeconomy, Queensland University of Technology (QUT), Brisbane, QLD 4000, Australia; peter.waterhouse@qut.edu.au; 2 ARC Centre of Excellence for Plant Success in Nature and Agriculture, Queensland University of Technology (QUT), Brisbane, QLD 4000, Australia

**Keywords:** biopharming, vaccines, viruses, viral vectors, *Nicotiana benthamiana*, COVID-19, plant-based biologics production

## Abstract

Severe virus outbreaks are occurring more often and spreading faster and further than ever. Preparedness plans based on lessons learned from past epidemics can guide behavioral and pharmacological interventions to contain and treat emergent diseases. Although conventional biologics production systems can meet the pharmaceutical needs of a community at homeostasis, the COVID-19 pandemic has created an abrupt rise in demand for vaccines and therapeutics that highlight the gaps in this supply chain’s ability to quickly develop and produce biologics in emergency situations given a short lead time. Considering the projected requirements for COVID-19 vaccines and the necessity for expedited large scale manufacture the capabilities of current biologics production systems should be surveyed to determine their applicability to pandemic preparedness. Plant-based biologics production systems have progressed to a state of commercial viability in the past 30 years with the capacity for production of complex, glycosylated, “mammalian compatible” molecules in a system with comparatively low production costs, high scalability, and production flexibility. Continued research drives the expansion of plant virus-based tools for harnessing the full production capacity from the plant biomass in transient systems. Here, we present an overview of vaccine production systems with a focus on plant-based production systems and their potential role as “first responders” in emergency pandemic situations.

## 1. Introduction

Biopharming is the use of a living system as a host for the manufacture of non-natively produced, biologic drugs. Using living systems as bio-factories can allow for economical production of complex biologics at large scales that may not be possible or economically feasible with current in vitro synthesis technologies. The first instance of this practice was the use of the bacterial host, *Escherichia coli*, to produce insulin in 1978 by Genentech, which was later commercialized in 1982 [1]. This alleviated the need for harvesting insulin from natural biological sources such as dog and calf pancreases [2]. The next technological leap for biopharming was the adoption of eukaryotic cells as production hosts, which allowed for the production of more complex molecules with mammalian type post translational modifications. This technology was first commercialized by Genentech in 1987 by repurposing *E. coli* fermenters for Chinese Hamster Ovary (CHO) cell production of the anticoagulant Activase [3,4]. This technological development was a boon for commercialization and CHO cells were quickly adopted as the preferred large-scale production host for complex therapeutic molecules. In 2017, the monoclonal antibodies (mAb) market was valued at 123 billion USD with 87% of newly approved mAb products being produced in CHO cells [5]. Developments in CHO cell biologics production technology have generated an efficient platform that is regarded as the industry standard, commercially available kits advertise a human antibody titer of 3 g/L with some groups reporting titers of >5.8 g/L [6,7]. The widespread adoption of this technology platform has led to government agencies developing regulatory frameworks that narrowly fit cell suspension-based production systems [8], and has consequently created hurdles for technologies that do not fit this format. Eukaryotic cell suspensions can be considered the second iteration of biopharming technology following prokaryotic production systems; however, in the biopharming space, a universal biologics production system does not yet exist. There are alternative systems that could avoid the expensive fermentation infrastructure, complex culturing conditions and lengthy development timelines associated with CHO cells. Currently biologics are produced in bacterial, yeast, mammalian, avian, insect, and plant systems. Advantages and disadvantages of these systems have been extensively reviewed and continuous developments have increased the yield and quality of biologics to the benchmark set by mammalian production systems [9,10]. The biologics production space is mainly dominated by fermentation-based technologies which in many cases require a lead time of as much as 12 months to select clones, optimize culturing conditions, and reach production capacity [11]. Transgenic animals, whole plants and embryonated hen’s eggs (EHE) stand apart as non-fermentation-based biologics productions hosts that have been used for commercial production. In this category, whole plants require the lowest input costs for biomass amplification and give the greatest production flexibility when used in transient expression systems. The coronavirus disease 2019 (COVID-19) pandemic has created an immediate need for vaccines and therapeutics to mitigate the spread and lethality of this disease. This urgent requirement for medicine has prompted our analysis of the current biologics production systems and their respective capacities for expedited drug development and scaling to large scale production. The goal of this review is to analyze and contrast the range of biopharming systems available, with particular emphasis on plant-based platforms, in the context of emergency pandemic response.

## 2. Plant Biopharming

### 2.1. Development of Biologics Production Systems in Plants

The first recorded example of biopharming in plants was the production of chimeric human growth hormone via transgenic tobacco and sunflower by Barta et al in 1986 [12]. The low infrastructure cost and simple biomass amplification requirements associated with plants compared to fermentation-based systems spurred an immense amount of interest in the possibilities of using plants as cheap biofactories and, by using the appropriate crop species, edible vaccines. This was soon followed by efforts to demonstrate the capacity for scaling plant-based biologics production in fields by using stably transformed crop plants such as maize, barley, safflower, and rice as production hosts. Although this approach held promise, early adopters of the technology were challenged by public perception of genetically modified plants, transgenic plant containment issues and a regulatory system with no precedent for good manufacturing practice (GMP) pharmaceuticals produced in this system [8]. In the following decades further investigation into plant-based production systems has been explored in a wide cross section of the plant kingdom including microalgae, moss, sundews, pitcher plants [13], melon [14], tomato, carrot, lettuce, tobacco, *Nicotiana benthamiana*, corn, rice, wheat, soybean, barley, and sunflower. The first generation of commercial biologics production in plants was centered on whole transgenic plants [15]. Today, this landscape is occupied by both transgenic and transient whole plant production systems as well as cell-culture-based systems and plant-based cell free systems [16]. The first genetically engineered plant derived therapeutic approved by the FDA was produced by Protalix in 2012. Protalix Biotherapeutics of Israel uses a transgenic carrot cell suspension system to produce taliglucerase alfa, for treatment of Gaucher disease [17]. Their production system is bioreactor-based and claims to have lower initial investment and running costs compared to mammalian-based systems [18]. Though many plant systems have been investigated for biologics production the current mainstream production host choice is *Nicotiana benthamiana.* It is the core production host of many companies including Medicago (https://www.medicago.com), Kentucky BioProcessing (https://kentuckybioprocessing.com), PlantForm (https://www.plantformcorp.com), Icon Genetics (https://www.icongenetics.com/), iBio (https://www.ibioinc.com), CapeBio (https://capebiosa.com), Bioapp (http://bioapp.co.kr) and Leaf Expression Systems (https://www.leafexpressionsystems.com). *N. benthamiana* was embraced by the research community because of its high susceptibility to pathogens which made it an excellent system for the study of plant pathogen interactions [19]. This Australian native plant is thought to have adopted a life strategy of sacrificing pathogen defenses in favor of a hastened reproduction cycle. This remarkable susceptibility to infection, by viruses in particular, is thought to play a role in the plant’s amenability to genetic transformation and high level transient gene expression, making it an excellent protein production host [20]. Transient expression in *N. benthamiana* allows the production of recombinant products in days rather than the 3- to 6-month timeline necessary when developing stable transgenic plants. In a typical *N. benthamiana* transient expression protocol, plants are grown to 4–6 weeks old then infected with a strain of the plant pathogen *Agrobacterium tumefaciens* containing genes of interest (GOI). *A. tumefaciens* transfers multiple copies of the GOI expression cassette to *N. benthamiana* which the plant then expresses in infected cells, the GOI product will typically reach peak level following a 5- to 7-day period. Product recovery can be achieved by homogenizing plant material and purification by a combination of filtration and chromatography methods. This system has been refined for biologics production by knocking out glycotransferases causing plant specific N linked glycosylations as well as development of methodologies for incorporation human type N and O glycosylations [21,22,23]. These refinements allow for production of recombinant proteins with mammalian glycosylation profiles. Further improvements to this system are continually arising with the goals of increasing product yield and quality by modifying the plant host, the *A. tumefaciens* strain, the infection methodologies and the DNA expression vector system [24]. In recent years, viral vector systems have provided the highest boosts in product yield in this transient system.

### 2.2. Viral Expression Vectors in Plants

A clear example of biopharming found in nature is the virus, which is an obligate parasite by definition, specializing in host invasion and redirection of biological processes for its own proliferation. Viral infection can commandeer host protein production systems causing accumulation of viral particles to 10% of plant dry weight [25]. This figure is likely the highest production of non-native protein in plants and is seen as the theoretical upper limit for transient protein production. Viral components have become a mainstay in plant biotechnology since the discovery of the cauliflower mosaic virus promoter in 1985, which was found to direct constitutive gene expression in most plant tissues and resulted in the highest known transgene expression at the time [26]. The use of viral components was further expanded by repurposing viral RNA silencing suppressors, such as P19 or V2, which overcome the RNA silencing machinery of the plant and inhibit degradation of foreign RNA [27,28]. When viral RNA silencing suppressors were expressed simultaneously with a GOI it resulted in a 50-fold increase of target protein yields [29]. A landmark discovery was the demonstration that GOIs could be inserted into the viral genome taking advantage of virus mobility and proliferation [30]. This “whole virus” approach is considered the first generation of viral vectors, whereby a GOI is inserted as a viral coat protein fusion or in place of the viral coat protein and relies on native virus infectivity and replication for GOI protein production [31]. The utility of this first approach was limited by non-comprehensive leaf coverage, low yields and insert size limitations, as viruses were shown to quickly lose the inserted gene during passage [30,32]. The second generation of viral vectors dubbed “deconstructed viral vectors” remove unnecessary viral component such as the coat protein while maintaining 5′ and 3′ UTR and replicase components. Deconstructed viral vectors rely on Agrobacterium infection for delivery to plants and the viral components for amplification and spread of the transgenic nucleic acid from cell to cell [25]. Notable examples of deconstructed viral vectors are the tobacco mosaic virus (TMV) derived magnICON and TRBO systems, the cowpea mosaic virus (CPMV) derived pEAQ and various potato virus X (PVX) based systems (Table 1). The first iteration of the magnICON system allowed for larger insert sizes, comprehensive tissue coverage and target product yields as high as 40% of total soluble protein or 4 g per kg fresh weight, with subsequent iterations of the technology reaching levels as high as 5.5 g/kg fresh weight [33,34]. Improvements in the most common systems based on TMV, CPMV, and PVX are typically achieved by removing and/or shuffling viral components and combining then into single vectors [34]. A common limitation of deconstructed viral vectors is their capacity for only one gene of interest per vector, which can be problematic for the expression of multichain products such as antibodies. This can be resolved by co-infiltration with non-competing TMV and PVX based systems [35]. The derivation of viral vectors from viruses is a field under constant development with the goals of expanding plant host range, increasing target protein yields, discovery of viral systems that can work in concert and mitigating deleterious effects to the production host. For example, foxtail mosaic virus has recently been shown to give improved monocot transformation, increased product yields and greater insert carrying capacity over the more traditional barley strip mosaic virus and wheat streak mosaic virus based systems [36]. Viral expression systems have cemented their position as a key component for high yielding transient expression and are likely to be the cornerstone of any commercialization venture involving biopharming in plants. Transient expression with viral vectors in *N. benthamiana* is a modular system with a flexibility not seen in other complex biologics production systems.

## 3. Systems for Vaccine Manufacture

Viral outbreaks of the past decade have solidified the perspective that containment is best achieved by quick detection informing nonpharmaceutical interventions followed by vaccination [57]. During the influenza A (H1N1) pandemic of 2009, aside from issues related to vaccine sharing and proper distribution, one of the key failings was insufficient global vaccine production capacity and production speed, which was unable to mitigate the spread of the first wave of infection. This was primarily a result of reliance on egg-based vaccine manufacturing systems with slow production speeds [58]. In theory, with proper communication, virus spread can be halted primarily through testing, isolation, and contact tracing of infected individuals followed by vaccinations pre-empting viral transmission to new areas [59,60,61]. These strategies were not put into practice for the SARS-CoV-2 pandemic which has caused massive shut downs in many parts of the world and provided the public with an opportunity to learn about the duration of clinical trials for a vaccine candidate. The H1N1 and COVID-19 outbreaks have also highlighted gaps in the vaccine production pipeline. Since 1945, governments worldwide have been reliant on egg-based vaccine production which use EHE as a host to replicate viruses which are subsequently purified then inactivated or attenuated. While this system is proven and is still considered a primary failsafe for disease outbreaks, the drawbacks are obvious in the face of an outbreak requiring a reactive response. Production pipelines are limited by the quantity of fertilized eggs available; the subsequent processing requires 14 days and can provide 5–20 mg of virus per 100 eggs [62]. This is accomplished in the US by an annual investment of at least 57 million USD in farms at undisclosed locations under federal contract by the department of health and human services [63]. Viral amplification for vaccine production has also been ported to several mammalian cell lines including Madin-Darby Canine Kidney (MDCK) cells, Vero cells originating from African green monkey kidney cells, Medical Research Council cell strain 5 (MRC5) cells, and Wistar Institute WI-38 cells. Both MRC5 and WI-38 cells originate from human fetal lung tissue, which confers the advantage over EHE vaccines of having a reduced risk of vaccine inefficiency due to avian specific viral adaptation or virus selection during viral passage. Additionally, scalability is not bottlenecked by egg production [64,65]. A specific drawback for the use of MDCK and EHE based systems to respond to the COVID-19 pandemic is their inability to support the replication of SARS-CoV-1 and SARS-CoV-2 viruses [66]. This deficiency highlights the problem with relying on native viral amplification for vaccine production. Next generation vaccines are not made from natively amplified viruses but use specific recombinant viral peptides or virus like particles (VLPs), composed of viral structural proteins and/or membrane elements expressed and assembled in the production host. VLPs are structurally identical to wildtype viral particles but lack the genetic material required to replicate and, because they are not reliant on native virus infectivity for inoculation and amplification, they can be produced in a wider range of host organisms such as insect cell lines and plants. This production methodology offers the advantages of safety because no live virus is present during manufacture and there are greater scaling options due to the range of production hosts available. In many cases VLPs have been equivalent or superior in their ability to raise an immune response in mice as compared to live viruses [67]. The US Food and Drug Administration (FDA) currently has two VLP vaccines for protection against human papilloma virus. As of 24 April 2020, there are 97 vaccines licensed for use in the US by the FDA, 33 of which are derived from EHEs, 27 have components produced in mammalian cells, 5 contain components produced in yeast, and 3 contain components produced in insect cells (Table 2). For the 2019–2020 flu season the US will offer its first egg-free influenza vaccine. In Canada, Medicago Inc. has recently completed a phase 3 trial for a plant-made VLP quadrivalent flu vaccine, which is an important milestone for plant-made biologics [68,69]. As these next generation vaccines begin to penetrate the market, this new technology promises more precise protection as well as a wider range of production options.

### Plant Systems for Viral Outbreak Response

Despite a modest presence of products on the pharmaceutical market, plant biopharming systems have been demonstrated on several occasions to be effective biologics production hosts, with the full capacity to produce correctly folded and glycosylated therapeutic molecules. In 2001, the Blue Angel Project sponsored by the US Defense Advanced Research Projects Agency sought to address, “insufficient capability to provide vaccines against pandemics caused by new strains, as well as infections caused by intentional biothreats”, by demonstrating the vaccine production capabilities of plant-based systems, by 1. developing a hardened, high containment, self-sufficient plant-based pharmaceutical production facility; 2. building a facility with the capacity to manufacture 10 million doses of an H1N1 influenza vaccine in a single month; and 3. completing this project within an 18 month window [72]. This project demonstrated that the plant-based production systems were capable of quick vaccine production and have the production pace that would be required to quell an unexpected viral outbreak. It was successfully completed in different stages by Medicago Inc., Caliber Biotherapeutics Inc. (now iBio Inc.), Fraunhofer CMB and Kentucky BioProcessing Inc. These companies operate currently as producers of biologics with portfolios including various vaccines and/or antibodies for cancer therapies. Today, Medicago reports that it can deliver mass quantities of a novel flu vaccine in a three-month timeline [73]. In 2014, The production speed of this system was demonstrated when Kentucky BioProcessing was able to quickly produce an Ebola antibody cocktail called Zmapp, developed by Mapp Biopharmaceutical, that had been granted emergency compassionate approval for human use [74]. This product, which is administered at 50 mg/kg, was produced in sufficient quantities to be used for the treatment of six people infected with Ebola, five of whom recovered. More recently, Medicago was able to produce VLP vaccine candidates 20 days after having access to the COVID-19 S protein sequence [75]. Although the long duration of clinical trials cannot be avoided, emergency governmental authorization to overlap clinical trials can shorten time to deployment for vaccines; making vaccine development and production timelines the bottlenecks prolonging the time to deployment [76].

The ability of plant-based biologics production facilities to quickly shift production pipelines for emergency manufacturing runs could be a great asset for pandemic situations and should be considered as an added value of these facilities by government sponsors. As seen in previous emergency pandemic responses, nations with vaccine production capabilities have had difficulties distributing vaccines to other countries without incentive [58]. This lack of vaccine sharing is likely to be repeated in the COVID-19 pandemic considering the economic consequences that this pandemic has brought already. These situations exemplify the need for decentralized biologics production lines to provide security for coming pandemics. With a comparatively low infrastructure cost, estimated at <50% of the cost of fermentation-based systems, plant-based biologics platforms make local vaccine and therapeutic production a more attainable goal for countries currently lacking pharmaceutical industry [77].

## 4. Conclusions and Future Perspectives

The immediate need for biologics in response to COVID-19 and the perceived lack of infrastructure to fill this demand has prompted analysis, by several groups, of how plant-based production systems can fill this need [78,79,80,81]. The ability of plant-based production systems to quickly pivot production to a variety of different target molecules and quickly produce large quantities at low cost is advantageous for pandemic response [73]. Indeed, several plant-based biologics manufacturers, using transient *N. benthamiana* expression systems, have initiated production of COVID-19-related products. Medicago and Kentucky BioProcessing have vaccines in clinical trial stages and iBio has 2 vaccine candidates and a therapeutic product currently in pre-clinical development. CapeBio and PlantForm Corporation are currently developing kits for COVID-19 testing and Leaf Expression Systems is producing viral proteins to support COVID-19 research and development. These corporations should be commended for their reactivity to the situation, and perhaps signal that plant-based platforms are now sufficiently developed to be a mainstream part of the plan to combat future outbreaks.

At the time of writing, there are 48 COVID-19 vaccines in the human trial phase, worldwide, with projected public release in early to mid-2021. Assuming a typical flu dosage of 45 ug per person, 45 g of vaccine will be required for 1 million people, which would scale to 351 kg for the world population. In addition to the need for vaccinations, there is also a requirement for therapeutic antibodies for those infected with COVID-19. In a recent review, Tusé et al. [78] estimated that the capacity of all mammalian cell fermentation facilities, worldwide, would be able to fulfill only 50% of this demand in one year, not including development time and assuming a low dose therapy (1 g per person). Current projections for vaccine release discuss prioritization of population segments for initial distribution, indicating a foreseen limitation in supplies. The COVID-19 pandemic will be resolved through a combined effort of different production systems to manufacture vaccines for public immunization as well as therapies for those infected. This situation has provided an opportunity to evaluate pandemic response systems globally and will be looked to in coming years for insight on the design of systems that can adequately respond to future outbreaks that are sure to come.

## Figures and Tables

**Table 1:** Example of plant viruses used as viral expression vectors and their selected applications.

Virus	Genome	Production Host	System, Comment, Reference	
Alfalfa mosaic virus (Alfamovirus)	I (+) ssRNA	*N. benthamiana*	VLPs/CP [37,38]	
Bamboo mosaic virus (Potexvirus)	F (+) ssRNA	*N. benthamiana,* *Chenopodium. quinoa*	Full length viral vectors [39]	
Beet Curly top virus (Curtovirus)	T-I (+) ssDNA	*N. benthamiana*	Deconstructed viral vectors [40,41,42]	
Bean yellow dwarf virus (Mastrevirus)	T-I (+) ssDNA	*N. benthamiana,* *Nicotiana tabacum,* lettuce	Deconstructed viral vectors/VLPs [39,40,42,43]	
Brome mosaic virus (Bromovirus)	I (+) ssRNA	Barley	1st plant RNA virus/VLPs [39,43]	
Beet necrotic yellow vein virus (Benyvirus)	RS (+) ssRNA	*N. benthamiana,* *C. quinoa*	Deconstructed viral vectors [44]	
Bean pod mottle virus (Comovirus)	I (+) ssRNA	Soybean, *P. sativum*	Deconstructed viral vectors [45]	
Barley stripe mosaic virus (Hordeivirus)	RS (+) ssRNA	Black-grass	Deconstructed viral vectors [46,47]	
Cauliflower mosaic virus (Caulimovirus)	I dsDNA	*Brassica rapa*	1st viral vector (Constitutive promoter)/Full length and deconstructed viral vectors [39]	
Catharantus yellow mosaic virus (Begomovirus)	T I (+) ssDNA	*Catharanthus roseus*	Deconstructed viral vectors [47]	
Cucumber green mottle mosaic virus (Tobamovirus)	RS (+) ssRNA	Muskmelon	Full length viral vectors [48]	
Cucumber mosaic virus (Cucumovirus)	I (+) ssRNA	*N. benthamiana*	Deconstructed viral vectors/VLPs [37,39,49]	
Cowpea mosaic virus (Comovirus)	I (+) ssRNA	*Vigna unguiculata*	1st virus applied as an epitope presentation system/Full length and deconstructed viral vectors/ VLPs [37,50]	
Citrus tristeza virus (Closterovirus)	F (+) ssRNA	Citrus trees	Deconstructed viral vectors [25]	
Foxtail mosaic virus (Potexvirus)	F (+) ssRNA	Maize, wheat, black-grass	Deconstructed viral vectors [36]	
Hibiscus chlorotic ringspot virus (Betacarmovirus)	I (+) ssRNA	Kenaf leaves	VLPs [39]	
Odontoglossum ringspot virus (Tobamovirus)	RS (+) ssRNA	*N. benthamiana*	Deconstructed viral vectors (hybrid with TMV) [25,39]	
Papaya mosaic virus (Potexvirus)	RS (+) ssRNA	*E. coli*	VLPs [39]	
Pea early browning virus (Tobravirus)	RS (+) ssRNA	*N. benthamiana*	Deconstructed viral vectors [36]	
Pepper ringspot virus (Tobravirus)	RS (+) ssRNA	*N. benthamiana*	Deconstructed viral vectors [49]	
Plum pox potyvirus (Potyvirus)	F R-S (+) ssRNA	*Nicotiana clevelandii*	Full length and deconstructed viral vectors [37,40,49,51]	
Potato virus X (Potexvirus)	F (+) ssRNA	*N. benthamiana*	Full length and deconstructed viral vectors/VLPs [37,50]	
Sun hemp mosaic virus (Tobamovirus)	RS (+) ssRNA	*N. benthamiana,* cowpea, lentil	Deconstructed viral vectors [52]	
Tomato bushy stunt virus (Tombusvirus)	I (+) ssRNA	*N. benthamiana, Nicotiana excelsiana*	Deconstructed viral vectors [37,50]	
Tobacco etch virus (Potyvirus)	RS (+) ssRNA	*Medicago trunculata*	Full length viral vectors [51]	
Tomato golden mosaic virus (Begomovirus)	T I (+) ssDNA	*N. benthamiana*	Deconstructed viral vectors [39]	
Tobacco mild green mosaic virus (Tobamovirus)	RS (+) ssRNA	*N. benthamiana*	Deconstructed viral vectors [52]	
Tobacco mosaic virus (Tobamovirus)	RS (+) ssRNA	*N. benthamiana, N. excelsiana*	Full length and deconstructed viral vectors/VLPs [37,39,49]	
Tomato mosaic virus (Tobamovirus)	RS (+) ssRNA	*N. tabacum*	Deconstructed viral vectors (hybrid with TMV) [39]	
Triticum mosaic virus (Tritimovirus)	F (+) ssRNA	Wheat, maize	Deconstructed viral vectors [53]	
Tobacco rattle virus (Tobravirus)	RS (+) ssRNA	*N. benthamiana*	Deconstructed viral vectors [54]	
Turnip vein-clearing virus (Tobamovirus)	RS (+) ssRNA	*N. benthamiana*	Deconstructed viral vectors (hybrid with TMV) [39]	
Tobacco yellow dwarf virus (Mastrevirus)	T I (+) ssDNA	*N. tabacum*	Deconstructed viral vectors [42]	
Wheat dwarf virus (Mastrevirus)	T I (+) ssDNA	*Triticum monococcum*	Deconstructed viral vectors [55]	
Turnip yellow mosaic virus (Tymovirus)	I (+) ssRNA	Cabbage	VLps [39]	
Wheat streak mosaic virus (Tritimovirus)	F (+) ssRNA	Wheat, maize	Deconstructed viral vectors [53]	
Zucchini yellow mosaic virus (Potyvirus)	F R-S (+) ssRNA	Squash, melon cucumber	Deconstructed viral vectors (particle bombardment) [56]	

I: Icosahedral, F: Filamentous, T: twinned, RS: rod-shaped, VLPs: Virus Like Particles, CP: Coat Protein.

**Table 2:** European Medicines Agency (EMA) and FDA Approved Vaccines 2020 [70,71].

Vaccine Indication and Number Approved by EMA; FDA	Production System(s) Associated with Vaccine	Type of Vaccines
Adenovirus Type 4 and Type 7 Vaccine, (0;1)	WI-38 human diploid cells	Live virus
Anthrax Vaccine (0;1)	*Bacillus anthracis*	Protective antigen protein from cell filtrates
BCG Vaccine (0;2)	*Mycobacterium bovis*	Attenuated bacteria
Cholera Vaccine (2;1)	*Vibrio cholera*	Attenuated bacteria
Dengue Vaccine (1;1)	Vero Cells	Live virus
Diphtheria and/or Tetanus and/or Acellular Pertussis and/or Hepatitis B and/or Polio and/or Hemophilus b and/or Vaccine (6;17)	*Corynebacterium diphtheriae, Clostridium tetani, Bordetella pertussis*, vero cells, *Saccharomyces cerevisiae, Haemophilus influenzae* type b and *Neisseria meningitidis*	Toxoids, antigens, inactivated virus, outer membrane protein, recombinant protein and capsular polysaccharide
Ebola Zaire Vaccine (3;1)	Vero cells	Live recombinant viral vaccine
Hepatitis A and/or Hepatitis B Vaccine (5;6)	MRC-5 human diploid cells, *Hansenula polymorpha* and *S. cerevisiae*	Inactivated virus, recombinant protein and VLP
Human Papillomavirus Vaccine (3;3)	*S. cerevisiae* and Baculovirus in *Trichoplusia ni* cells	VLP
Influenza A H1N1 Vaccine (1;7)	Embryonated chicken eggs	Inactivated virus and live virus
Influenza A H5N1 Vaccine (6;2)	Embryonated chicken eggs and MDCK cells	Inactivated virus
Influenza Vaccine (3;29)	Embryonated chicken eggs, MDCK cells and Sf9 cells	Inactivated virus and recombinant HA protein
Japanese Encephalitis Virus Vaccine (1;1)	Vero cells	Inactivated virus
Measles, Mumps, and Rubella Virus Vaccine (1;1)	Chick embryo cell culture and WI-38 human diploid cells	Attenuated and live virus vaccine
Measles, Mumps, Rubella and Varicella Virus Vaccine (1;1)	Chick embryo cell culture, WI-38 human diploid cells and MRC-5 human diploid cells	Attenuated and live virus vaccine
Meningococcal Vaccine (3;6)	*C. diphtheriae, N. meningitidis, E. coli* and *C. tetani*	Capsular polysaccharide, capsular polysaccharide toxoid conjugate, recombinant protein and outer membrane vesicle
Pneumococcal Vaccine (2;2)	*Streptococcus pneumoniae* and *C. diphtheriae*	Capsular polysaccharide and capsular polysaccharide protein conjugate
Rabies Vaccine (Human) (0;2)	MRC-5 human diploid cells and chicken fibroblasts	Inactivated virus
Rotavirus Vaccine (2;2)	Vero cells	Live attenuated virus
Smallpox (1;1)	Vero cells	Live virus
Smallpox and Monkeypox Vaccine (0;1)	Chicken embryo fibroblasts	Live virus
Typhoid Vaccine (2;1)	*Salmonella typhi* Ty21a	Live attenuated virus, capsular polysaccharide
Varicella Virus Vaccine (0;1)	WI-38 human diploid cells, MRC-5 human diploid cells	Live attenuated virus
Yellow Fever Vaccine (1;1)	Living avian leukosis virus-free chicken embryos	Attenuated virus
Zoster Vaccine (2;2)	MRC-5 human diploid cells, CHO and *Salmonella minnesota*	Live attenuated virus and virus surface glycoprotein E (gE) antigen c
